# Characterization of cholesterol homeostasis in sphingosine-1-phosphate lyase-deficient fibroblasts reveals a Niemann-Pick disease type C-like phenotype with enhanced lysosomal Ca^2**+**^ storage

**DOI:** 10.1038/srep43575

**Published:** 2017-03-06

**Authors:** Hans Vienken, Nathalie Mabrouki, Katja Grabau, Ralf Frederik Claas, Agnes Rudowski, Nina Schömel, Josef Pfeilschifter, Dieter Lütjohann, Gerhild van Echten-Deckert, Dagmar Meyer zu Heringdorf

**Affiliations:** 1 Institut für Allgemeine Pharmakologie und Toxikologie, Klinikum der Goethe-Universität, Frankfurt am Main, Germany; 2 Institut für Klinische Chemie und Klinische Pharmakologie, Universitätsklinikum Bonn, Bonn, Germany; 3 Membranbiologie und Lipidbiochemie, Einheit des Life and Medical Sciences (LIMES) Instituts, Universität Bonn, Bonn, Germany

## Abstract

Sphingosine-1-phosphate (S1P) lyase irreversibly cleaves S1P, thereby catalysing the ultimate step of sphingolipid degradation. We show here that embryonic fibroblasts from S1P lyase-deficient mice (*Sgpl1*^−/−^-MEFs), in which S1P and sphingosine accumulate, have features of Niemann-Pick disease type C (NPC) cells. In the presence of serum, overall cholesterol content was elevated in *Sgpl1*^−/−^-MEFs, due to upregulation of the LDL receptor and enhanced cholesterol uptake. Despite this, activation of sterol regulatory element-binding protein-2 was increased in *Sgpl1*^−/−^-MEFs, indicating a local lack of cholesterol at the ER. Indeed, free cholesterol was retained in NPC1-containing vesicles, which is a hallmark of NPC. Furthermore, upregulation of amyloid precursor protein in *Sgpl1*^−/−^-MEFs was mimicked by an NPC1 inhibitor in *Sgpl1*^+/+^-MEFs and reduced by overexpression of NPC1. Lysosomal pH was not altered by S1P lyase deficiency, similar to NPC. Interestingly, lysosomal Ca^2+^ content and bafilomycin A1-induced [Ca^2+^]_i_ increases were enhanced in *Sgpl1*^−/−^-MEFs, contrary to NPC. These results show that both a primary defect in cholesterol trafficking and S1P lyase deficiency cause overlapping phenotypic alterations, and challenge the present view on the role of sphingosine in lysosomal Ca^2+^ homeostasis.

Sphingosine-1-phosphate (S1P) lyase catalyses the irreversible cleavage of S1P, which plays an important role in regulation of crucial cellular functions such as cell growth, survival, adhesion and migration^1^,^2^,^3^. S1P acts both as agonist at specific G-protein-coupled receptors (GPCR) and intracellularly^1^,^2^. The five S1P-GPCR couple differentially to G_i_, G_q_ and G_12/13_ proteins, and regulate for example lymphocyte trafficking, angiogenesis, vascular tone and permeability, and homeostasis of many tissues^2^,^4^. Pathophysiologically, S1P-GPCR play a role in autoimmunity, inflammation, fibrosis, and cancer, and they are appreciated as important pharmacological targets in these conditions^5^,^6^,^7^,^8^,^9^,^10^. The intracellular activities of S1P are less well understood. It has been suggested that intracellular S1P inhibits histone deacetylases (HDACs), acts as cofactor of the E3 ubiquitin ligase TRAF-2, plays a role in mitochondrial respiration by binding to prohibitin-2, and activates β-site amyloid precursor protein cleaving enzyme-1 (reviewed in ref. 1).

S1P lyase not just cleaves S1P, but catalyses the ultimate step of sphingolipid degradation, and thereby has an influence on cellular concentrations of sphingolipids further upstream of S1P^11^. The importance of S1P lyase becomes obvious when looking at the phenotype of mice lacking this enzyme: *Sgpl1*^−/−^-mice have a growth retardation and die within a few weeks after birth^12^,^13^. They suffer from multiple organ defects including malformations of the vasculature and the skeleton, and a kidney dysfunction^12^. Significant lesions were detected in histological sections of lung, heart, urinary tract and bone of these animals^13^. S1P lyase-deficient mice have a lymphopenia, which is explained by disruption of the blood-tissue S1P gradient essential for lymphocyte trafficking^11^,^13^,^14^. Furthermore, they have a pro-inflammatory phenotype with neutrophilia but impaired neutrophil recruitment into inflamed tissues^14^. Not only S1P, but also sphingosine and/or ceramide accumulate in serum and tissues of *Sgpl1*^−/−^-mice^13^,^14^,^15^. A thorough study on lipid profiles in liver and plasma of S1P lyase-deficient mice revealed that aside from sphingolipids, also total and free cholesterol, cholesterol esters, phospholipids, and triacylglycerol were strongly elevated in the serum of *Sgpl1*^−/−^-mice. Both low-density lipoprotein (LDL) and high-density lipoprotein (HDL) cholesterol were elevated^16^. In the liver of *Sgpl1*^−/−^-mice, S1P reached concentrations as high as 1 nmol/mg, and sphingosine reached 3 nmol/mg protein, while levels of ceramide and sphingomyelin were significantly albeit less enhanced. Also triacylglycerol and cholesterol esters, but not free cholesterol, were elevated in the mouse livers^16^. This pioneering study thus demonstrated a link between S1P lyase deficiency and homeostasis of cholesterol and triglycerides. Mechanistically, an involvement of peroxisome proliferator-activated receptor-γ was suggested^16^, however, the ongoing events could not be fully elucidated.

Aim of the present study was to analyse the link between S1P/sphingosine and cholesterol homeostasis using embryonic fibroblasts from S1P lyase-deficient mice. Fibroblasts have turned out to be useful for studying cellular cholesterol homeostasis, and much of the work on Niemann-Pick disease type C (NPC) has been performed in fibroblasts, including CHO cells and patient skin fibroblasts. *Sgpl1*^−/−^-MEFs have been used for analysis of intracellular activities of S1P and therefore they are well-characterized. *Sgpl1*^−/−^-MEFs proliferate well; in the absence of serum, they grow faster than their wild-type counterparts^17^. They are resistant to chemotherapy-induced apoptosis probably due to upregulation of both multidrug transporters and Bcl-2/Bcl-xL^17^,^18^. Furthermore, they are characterized by alterations in cellular Ca^2+^ homeostasis with enhanced increases in cytosolic Ca^2+^ concentrations ([Ca^2+^]_i_) in response to agonists, elevated basal [Ca^2+^]_i_ and enhanced Ca^2+^ storage in thapsigargin-sensitive stores^19^. S1P accumulated by about 6-fold and sphingosine by about 2-fold in these cells^19^, but we did not observe a significant increase in cellular ceramides (data not shown). Interestingly, measurements of S1P and sphingosine in nuclear preparations of S1P lyase-deficient MEFs revealed that the accumulating S1P was nearly fully associated with the nuclei. In contrast, sphingosine levels were identical in nuclei of *Sgpl1*^+/+^- and *Sgpl1*^−/−^-MEFs^20^, suggesting that this lipid accumulated elsewhere in the cells. Furthermore, HDAC activity was decreased and expression of HDAC1 and HDAC3, but not HDAC2, was reduced in S1P lyase-deficient MEFs^20^. The reduced HDAC activity contributed to the phenotype of Ca^2+^ homeostasis in these cells^20^.

We show here that in S1P lyase-deficient MEFs, free cholesterol is trapped in NPC1-expressing vesicles, leading to activation of sterol regulatory element binding protein-2 (SREBP-2), upregulation of the LDL receptor and enhanced cholesterol uptake in the presence of serum. Interestingly, in contrast to NPC, lysosomal Ca^2+^ storage was not reduced but significantly enhanced in *Sgpl1*^−/−^-MEFs. These results shed new light on the interplay between S1P/sphingosine and cholesterol, and on the regulation of lysosomal Ca^2+^ homeostasis.

## Results

### Enhanced cholesterol content in S1P lyase-deficient MEFs in the presence of serum

**Figure 1:**
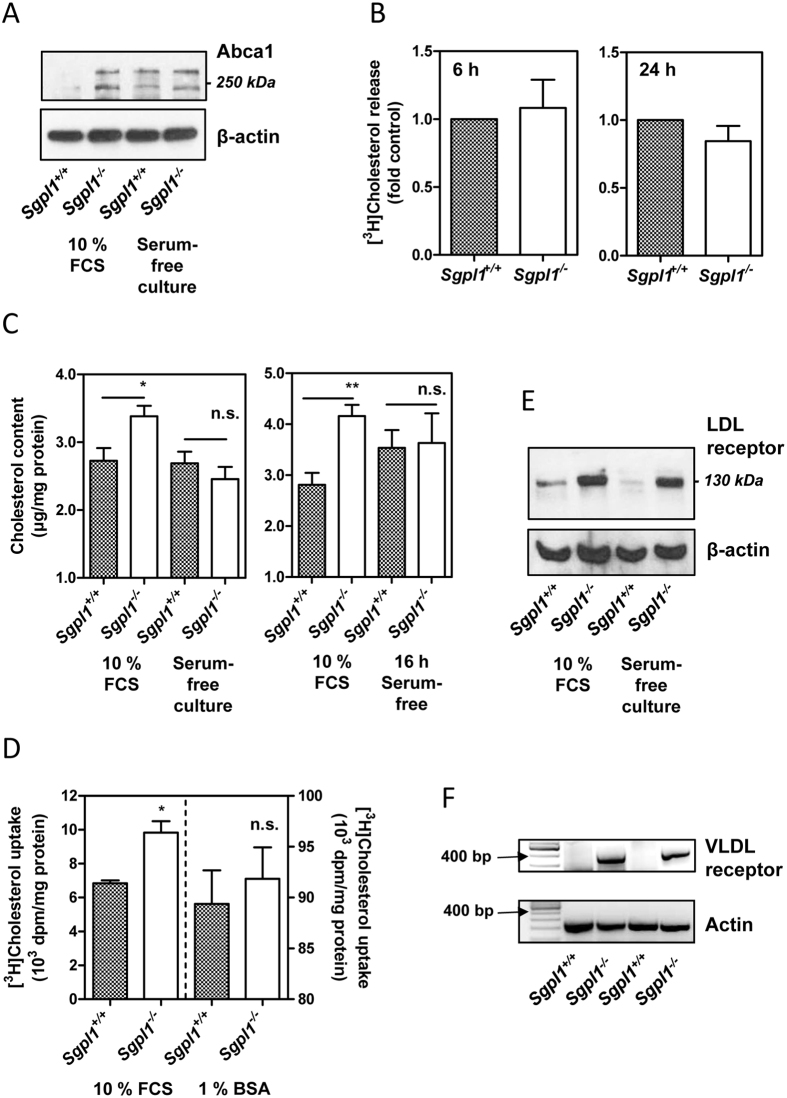
Content, uptake and release of cholesterol in S1P lyase-deficient MEFs. (**A**) Expression of Abca1 was analyzed by Western blotting. The cells were grown either in the presence of 10% FCS or in serum-free medium supplemented with growth factors. Representative blot showing glycosylated and unglycosylated forms of Abca1. (**B**) Cholesterol release was measured after loading with [^3^H]cholesterol in the presence of 10% FCS. The cells were incubated for 6 or 24 h, respectively, in serum-free medium supplemented with 1% BSA and 250 ng/ml ApoA-I as acceptors of cholesterol. Means ± SEM; n = 3. (**C**) Cholesterol content was measured with the Amplex Red assay. In the first series of experiments, the cells were cultured either in the presence of 10% FCS or in serum-free medium supplemented with growth factors (left panel; n = 8). In the second series, they were grown in the presence of 10% FCS or set to serum-free medium for 16 h before measurements (right panel; n = 7). Means ± SEM; *p < 0.05; **p < 0.01; n.s., not significant (t-test). (**D**) Uptake of [^3^H]cholesterol was measured after 2 h of incubation in medium containing either 10% FCS or 1% BSA. Means ± SEM; n = 3; *p < 0.05; n.s., not significant (t-test). (**E**) Expression of the LDL receptor was analyzed by Western blotting. The cells were grown either in the presence of 10% FCS or in serum-free medium supplemented with growth factors. Representative blot. (**F**) Expression of the VLDL receptor was analyzed by classical PCR. Shown are PCR fragments (45 cycles) of two independent experiments.

In the course of analysing the expression of ABC transporters in S1P lyase-deficient MEFs, we had noticed that among diverse transporters that were upregulated in comparison to wild-type cells, there was ATP-binding cassette transporter-A1 (Abca1)^18^. Indeed, not only Abca1 mRNA but also the glycosylated and non-glycosylated forms of the Abca1 protein were strongly upregulated in S1P lyase-deficient MEFs when the cells were kept in the presence of 10% FCS (Fig. 1A). Furthermore, Abca1 was upregulated in wild-type cells when the cells were grown in serum-free medium and not much further induced in S1P lyase-deficient cells under these conditions (Fig. 1A). Since Abca1 has been described as transporter for both cholesterol and S1P^21^,^22^, we hypothesized that by upregulating this protein, the cells attempted to reduce intracellularly accumulating S1P at the expense of cholesterol. However, release of [^3^H]cholesterol in the presence of bovine serum albumin (BSA) and apolipoprotein A-I (ApoA-I) as acceptors was not altered in S1P lyase-deficient MEFs (Fig. 1B). This matches the surprising lack of enhanced S1P secretion in these cells^18^ and might be due to lack of proper insertion of the transporters into the plasma membrane which was evident at least for Abcb1 and Abcc1^18^. When we measured intracellular cholesterol with the Amplex Red assay, we noticed that in the presence of 10% serum, S1P lyase-deficient MEFs contained significantly higher levels of cholesterol than wild-type cells (Fig. 1C). This was not the case when the cells were grown in serum-free medium or set to serum-free medium for 16 h before analysis (Fig. 1C). Furthermore, uptake of [^3^H]cholesterol was enhanced in S1P lyase-deficient cells in the presence of serum, but not when the lipid was offered in a complex with BSA, although the uptake was highly efficient under this condition (Fig. 1D). In support of the hypothesis that [^3^H]cholesterol was preferentially taken up by the knockout cells when offered in the presence of plasma lipoproteins, both the LDL receptor (protein) and very low-density lipoprotein (VLDL) receptor (mRNA) were upregulated in S1P lyase-deficient MEFs (Fig. 1E,F). In summary, we observed that S1P lyase-deficient MEFs had an elevated content of cholesterol when grown in the presence of serum, and that this was due to enhanced uptake of cholesterol-containing lipoproteins.

### Dysregulation of intracellular cholesterol distribution in S1P lyase-deficient MEFs

**Figure 2:**
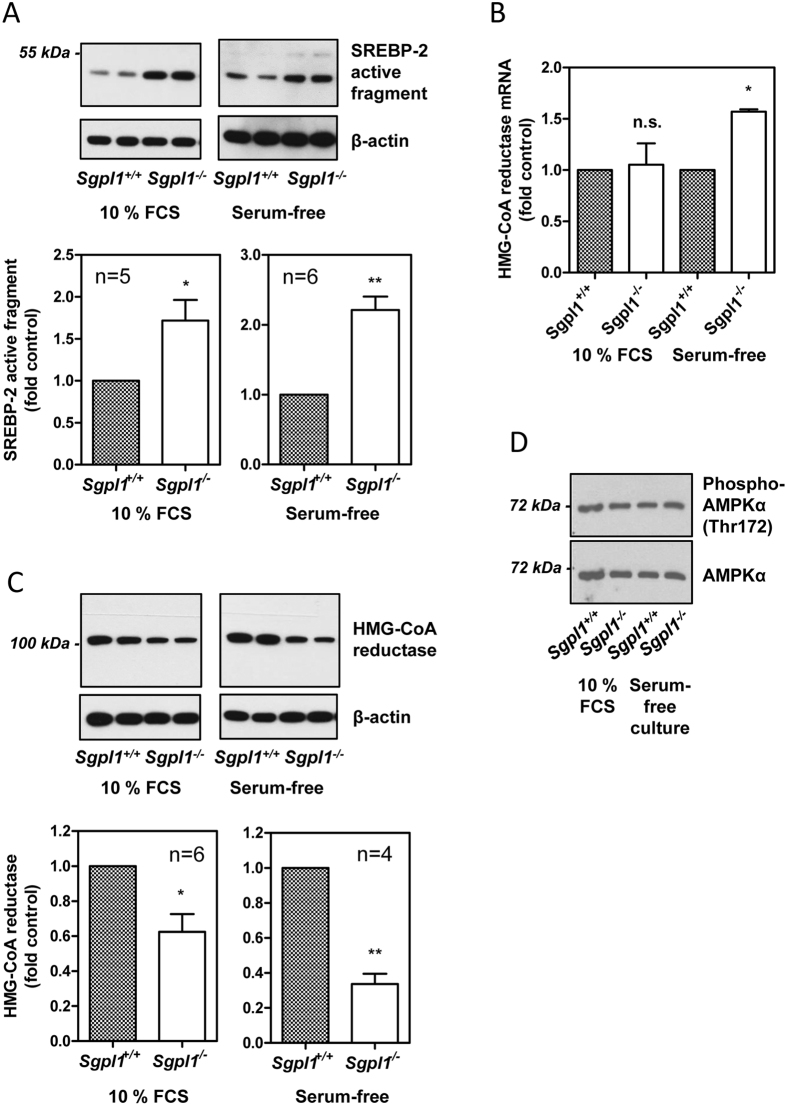
Activation of SREBP-2 and expression of HMG-CoA reductase. (**A**) Proteolytic activation of SREBP-2 was analyzed by Western blotting. The cells were grown in the presence of 10% FCS or set to serum-free medium for 16 h before measurements. Upper panels: representative blots. Lower panels: Quantification showing means ± SEM. (**B**) mRNA expression of HMG-CoA reductase was analyzed by quantitative PCR. Shown are means ± SEM of n = 3 (10% FCS) or n = 5 (16 h serum-free medium) independent experiments. (**C**) HMG-CoA reductase protein expression was analyzed by Western Blotting. The cells were grown in the presence of 10% FCS or kept for 16 h in serum-free medium. Upper panels: representative blots. Lower panels: Quantification showing means ± SEM. (**D**) Western blot analysis of phospho-AMPKα (Thr172) and AMPKα in MEFs grown in the presence of 10% FCS or in serum-free medium supplemented with growth factors (representative blot). *p < 0.05; **p < 0.01; n.s., not significant in one-sample t-test.

There are two key transcription factors which regulate the expression of genes involved in the maintenance of cellular cholesterol homeostasis, liver X receptor and SREBP-2^23^,^24^. While liver X receptor mRNA was not expressed, SREBP-2 mRNA was present in both *Sgpl1*^+/+^- and *Sgpl1*^−/−^-MEFs (data not shown). SREBP-2 is activated when low cholesterol levels at the ER, sensed by SREBP-cleavage activating and insig proteins, lead to proteolytic cleavage of SREBP-2^23^. The release of SREBP-2′s transcriptionally active fragment then induces the expression of genes such as the LDL receptor and 3-hydroxy-3-methyl-glutaryl-coenzyme A (HMG-CoA) reductase, with the aim of compensating cellular cholesterol shortage^23^,^24^. The active fragment of SREBP-2 could be identified by Western blotting in both *Sgpl1*^+/+^- and *Sgpl1*^−/−^-MEFs (Fig. 2A). Interestingly, there was significantly more SREBP-2 cleavage in S1P lyase-deficient MEFs, both in the presence of 10% FCS and after 16 h of serum-free medium (Fig. 2A). This was surprising in the light of similar overall cholesterol content in serum-free conditions and even higher overall cholesterol content in the presence of 10% FCS (as shown in Fig. 1C), but could explain the upregulation of Abca1 and the LDL receptor in these cells. Activated SREBP-2 also induces expression of the key enzyme of cholesterol biosynthesis, HMG-CoA reductase. Indeed, HMG-CoA reductase mRNA was upregulated under serum-free conditions, although it remained unaltered in the presence of 10% FCS (Fig. 2B), suggesting that the observed increase in cholesterol uptake by *Sgpl1*^−/−^-MEFs in the presence of serum was able to compensate at least partially the lack of cholesterol at the ER. On the other hand, the protein levels of HMG-CoA reductase were significantly decreased in the knockout MEFs (Fig. 2C), indicating a posttranscriptional downregulation of this enzyme. Finally, we analysed the phosphorylation of AMP-activated kinase (AMPK)-α at Thr172 as indicator for the cellular energy state and as regulator of HMG-CoA reductase activity^25^. However, despite the elevated basal [Ca^2+^]_i_ in S1P lyase-deficient MEFs^19^, which via Ca^2+^/calmodulin-dependent kinase kinase can lead to phosphorylation of AMPKα at Thr172^25^, there was no difference in phospho-AMPKα or total AMPKα between *Sgpl1*^+/+^- and *Sgpl1*^−/−^-MEFs (Fig. 2D).

**Figure 3:**
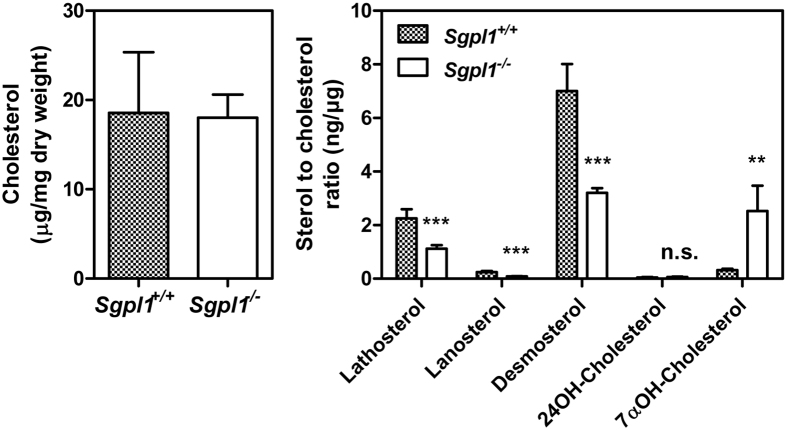
Cellular levels of cholesterol, non-cholesterol sterols and oxysterols. The measurements were performed by GC-flame ionization detection for cholesterol and GC-mass spectrometry detector-selected ion monitoring for sterols and oxysterols except cholesterol in MEFs that had been kept in 0.3% FCS. The values are means ± SD, n = 4. **p < 0.01; ***p < 0.001; n.s., not significant in t-test.

A detailed analysis of cellular sterols was performed by gas chromatography (GC)^26^,^27^ in MEFs that had been kept in 0.3% FCS. In the presence of this low amount of serum, total cholesterol content was similar in *Sgpl1*^+/+^- and *Sgpl1*^−/−^-MEFs (Fig. 3). In agreement with reduced HMG-CoA reductase protein expression, cholesterol precursors such as lathosterol, lanosterol and desmosterol were strongly reduced in *Sgpl1*^−/−^-MEFs (Fig. 3). Interestingly, of the oxysterols, 7α-hydroxycholesterol, in MEFs produced by autoxidation, was elevated by about 8-fold in the knockout cells, while the abundance of 24-hydroxycholesterol, formed by CYP46A1, was generally low but not different in *Sgpl1*^+/+^- and *Sgpl1*^−/−^-MEFs (Fig. 3)^28^,^29^.

**Figure 4:**
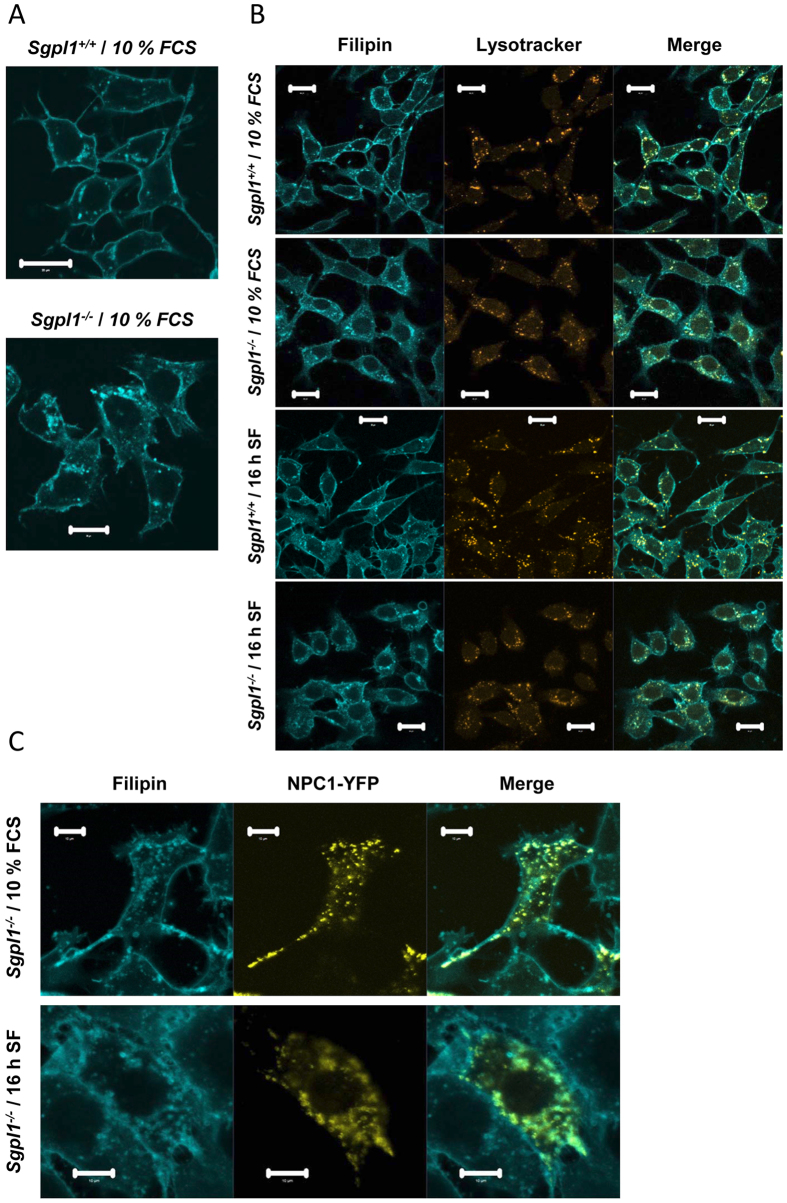
Subcellular distribution of free cholesterol and colocalization with lysosomes and NPC1. The cells were grown in the presence of 10% FCS or set to serum-free medium for 16 h before measurements (16 h SF). (**A**) For staining of free cholesterol, the cells were fixed with paraformaldehyde and stained filipin III (cyan). Bars, 20 μm. (**B**) For concomitant staining of lysosomes, the cells were incubated with LysoTracker Red DND-99 (orange) before fixing with paraformaldehyde and staining with filipin. Bars, 20 μm. (**C**) The cells were transfected with NPC1-YFP before fixing with paraformaldehyde and staining with filipin. Bars, 10 μm.

Thus, SREBP-2 cleavage was enhanced in S1P lyase-deficient MEFs, although the overall cellular cholesterol content was not altered in the absence of serum and even increased in the presence of serum. Since this was suggestive of a defect in intracellular cholesterol distribution, we analysed the localization of free cholesterol by staining with filipin. In wild type cells, a strong staining of the plasma membrane was prevailing, with some additional staining at intracellular vesicles (Fig. 4A,B). In contrast, in S1P lyase-deficient MEFs, there was a strong filipin fluorescence at intracellular vesicles, and a relatively weaker staining of the plasma membrane (Fig. 4A,B). This pattern was more pronounced in the presence of 10% FCS than in serum-free conditions (Fig. 4A,B). There was a partial overlap of filipin staining with lysotracker fluorescence in both cell types, indicating that cholesterol-containing vesicles were at least in part acidic compartments such as lysosomes (Fig. 4B). A key regulator of intracellular distribution of cholesterol, after endocytosis of LDL particles, from the endo-lysosomal compartment to other cellular membranes such as plasma membrane and ER, is NPC1^30^. Indeed, filipin staining co-localized strongly with the fluorescence of overexpressed NPC1-YFP in S1P lyase-deficient MEFs, suggesting that cholesterol was contained in NPC1-expressing compartments in these cells (Fig. 4C).

Taken together, SREBP-2 activation was enhanced and SREBP-2 target genes were upregulated in S1P lyase-deficient MEFs, leading to enhanced cholesterol uptake which compensated for decreased HMG-CoA reductase protein expression and cholesterol biosynthesis. The enhanced SREBP-2 activation, indicative of a shortage in cholesterol at the ER despite unchanged or even elevated overall cellular cholesterol levels, was due to a disturbance of intracellular cholesterol distribution, with the lipid being trapped in NPC1-containing compartments.

### Indicators for an impaired function of NPC1 in S1P lyase-deficient MEFs

**Figure 5:**
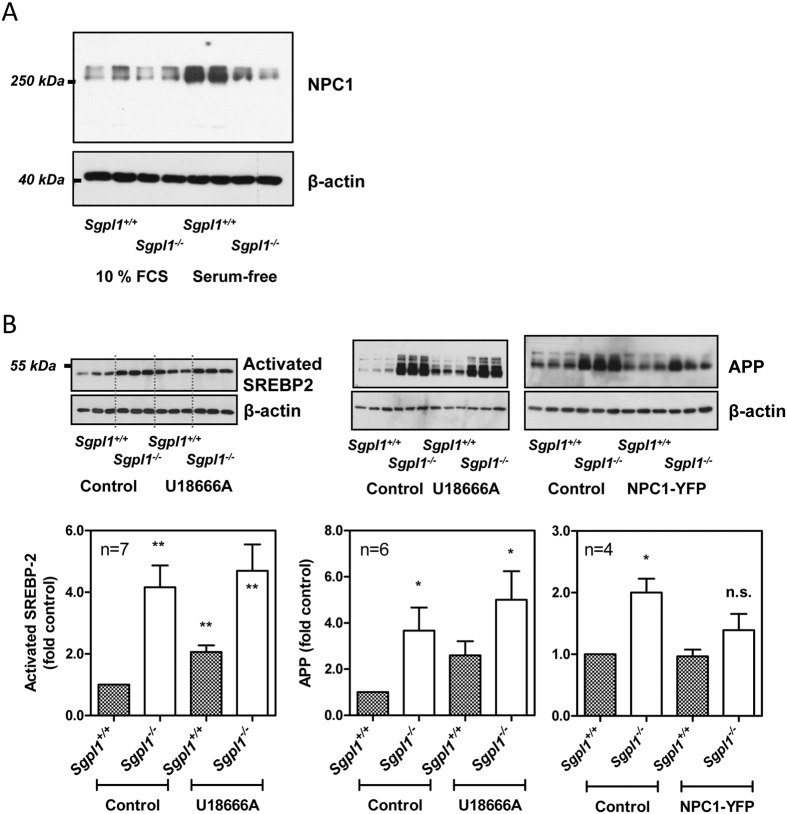
Expression of NPC1 and NPC marker proteins. (**A**) The cells were grown in the presence of 10% FCS or set to serum-free medium for 16 h before preparation of cell lysates and analysis of NPC1 expression by Western blotting. Representative blot. (**B**) Influence of U18666A and NPC1-YFP overexpression on SREBP-2 activation and expression of APP. Incubation with 25 μM U18666A was performed for 16 h in serum-free medium. After transfection with NPC1-YFP, the cells were kept for 48 h in the presence of 10% FCS. Shown are representative blots and quantitative analyses with means ± SEM of the indicated number of independent experiments performed in duplicate or triplicate. *p < 0.05; **p < 0.01; n.s., not significant in one-sample t-test.

Impaired trafficking of cholesterol from the late endosomal/lysosomal compartment to the ER is a hallmark of NPC1 deficiency or dysfunction^31^,^32^. Therefore, we analyzed the expression of endogenous NPC1 in S1P lyase-deficient MEFs. However, NPC1 protein expression was not significantly different in *Sgpl1*^+/+^- and *Sgpl1*^−/−^-MEFs in the presence of 10% FCS (Fig. 5A), suggesting that the phenotype with activation of SREBP-2 and accumulation of free cholesterol in endosomal/lysosomal compartments in S1P lyase-deficient MEFs was rather due to an impairment of NPC1 function than to a lack of NPC1 overall protein. In agreement with previous reports which had shown a downregulation of NPC1 by addition of LDL, and an upregulation of NPC1 by binding of SREBP-2 to the NPC1 promoter^33^, NPC1 was strongly upregulated upon serum depletion in wild-type cells (Fig. 5A). Interestingly, the expression of NPC1 remained unaltered by serum depletion in S1P lyase-deficient cells (Fig. 5A). Thus, in serum-free medium, S1P lyase-deficient MEFs had significantly less NPC1 protein than wild-type cells (0.48 ± 0.10-fold; mean ± SEM; n = 3 independent experiments; p < 0.05), although SREBP-2 cleavage was more pronounced in the cells. This points towards a mechanism by which NPC1 induction or stability is impaired in S1P lyase-deficient MEFs in serum-free conditions, in addition to a potential functional impairment.

U18666A, which is an inhibitor of intracellular cholesterol trafficking and has recently been shown to directly bind and inhibit NPC1^34^, is often used to imitate the NPC1 mutant phenotype. In agreement with previous publications in other cell types^35^, U18666A enhanced the expression of SREBP-2 activated fragment in wild-type MEFs (Fig. 5B). In contrast, U18666A did not further increase SREBP-2 cleavage in the knockout MEFs (Fig. 5B). We also analysed whether U18666A had an influence on expression and activity of S1P lyase. Indeed, treatment of wild-type MEFs with U18666A induced a strong increase in *Sgpl1* mRNA, however, S1P lyase protein levels were not altered and S1P lyase activity was slightly but not significantly decreased by U18666A (Supplemental Fig. 1). One of the hallmarks of NPC1 dysfunction is enhanced expression of amyloid precursor protein (APP) and dysregulation of APP processing^36^. Interestingly, this phenotype is present also in S1P lyase-deficient cells^37^. In agreement with a role for NPC1 in regulation of APP in S1P lyase-deficient cells, U18666A increased the expression of APP in wild-type MEFs, while overexpression of NPC1-YFP, despite a low transfection efficiency of <10%, reduced APP levels in S1P lyase-deficient cells down to a level no more significantly different from control (Fig. 5B). Finally, we wondered whether overexpression of S1P lyase would counteract the effect of U18666A on sequestration of cholesterol. We had shown before that expression of YFP-tagged S1P lyase significantly reduced the elevated [Ca^2+^]_i_ and thapsigargin-induced [Ca^2+^]_i_ increases in *Sgpl1*^−/−^-MEFs^20^. However, U18666A-induced sequestration of cholesterol, analysed by filipin staining, was not different in cells expressing YFP-S1P lyase compared to non-transfected neighbouring cells (Supplemental Fig. 2).

### Lysosomal Ca^2+^ homeostasis in S1P lyase-deficient MEFs

**Figure 6:**
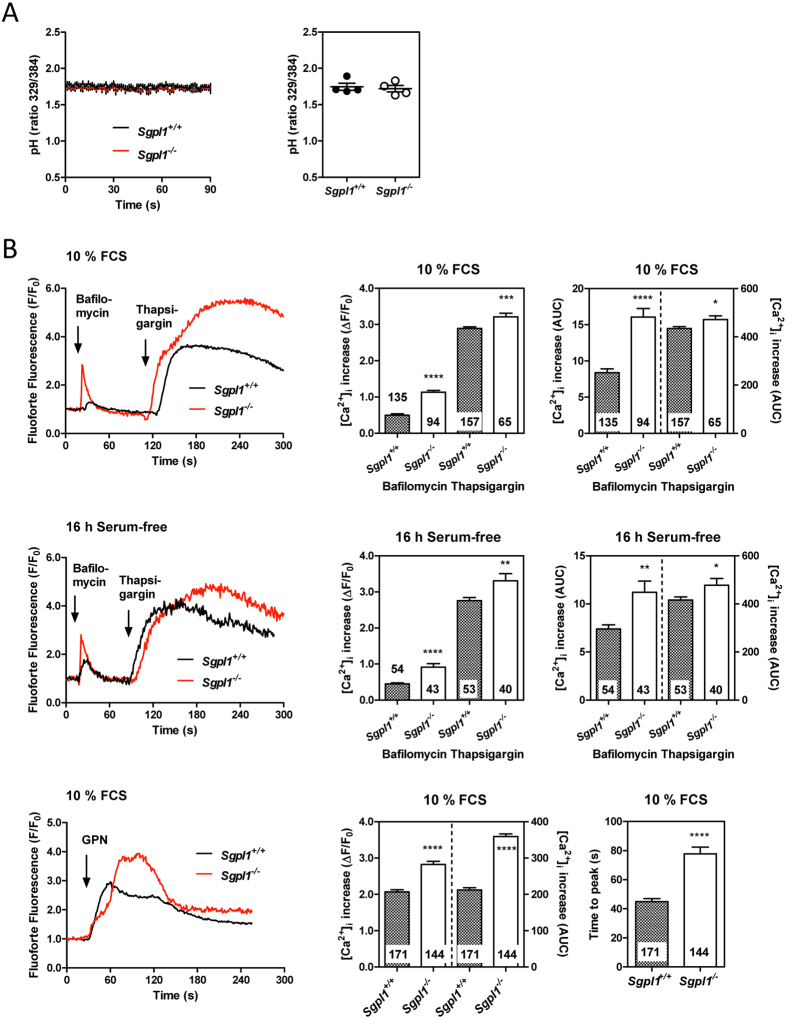
Lysosomal pH and Ca^2+^ release. (**A**) Relative measurements of lysosomal pH were performed in cell suspensions after loading with 100 nM LysoSensor Yellow/Blue DND-160. Excitation was altered between 329 and 384 nm while emission was recorded at 490 nm. Left, stability of pH during the measurements. Excitation ratios were recorded for ~90–120 s. Lines represent means ± SEM of individual samples (n = 12 each). Right, means from 4 independent experiments with 3 samples each of both *Sgpl1*^+/+^- and *Sgpl1*^−/−^-MEFs. The cells had been kept for 16 h in serum-free medium. (**B**) [Ca^2+^]_i_ increases were analyzed in MEFs grown on 8-well chambered coverslides and loaded with GFP-certified FluoForte. The cells were stimulated at the indicated time points with bafilomycin A1 (1 μM), thapsigargin (1 μM) or GPN (200 μM). Left, representative traces of [Ca^2+^]_i_ from single cells. Right, quantifications of peak [Ca^2+^]_i_ increases and AUCs. The values represent means ± SEM from the indicated number of individual cells. *p < 0.05; **p < 0.01; ***p < 0.001; ****p < 0.0001; t-test.

We furthermore addressed the question of lysosomal pH and Ca^2+^ content. Studies on cells deficient in NPC1 or expressing NPC1 mutants suggest that lysosomal pH is not altered in NPC, while Ca^2+^ storage and/or release of Ca^2+^ from acidic compartments is impaired by NPC1 dysfunction^38^,^39^,^40^. In fact, using the acidotropic and ratiometric pH sensor, LysoSensor Yellow/Blue DND-160, we did not detect a difference in lysosomal pH between *Sgpl1*^+/+^- and *Sgpl1*^−/−^-MEFs (Fig. 6A). We also measured the increase in [Ca^2+^]_i_ by the inhibitor of the vesicular H^+^ -ATPase, bafilomycin A1, in single MEFs loaded with the FluoForte Ca^2+^ sensor. In clear contrast to observations in NPC1 mutant cells, bafilomycin A1-induced peak [Ca^2+^]_i_ increases and areas under the curve (AUCs) were not reduced in *Sgpl1*^−/−^-MEFs, but significantly enhanced (Fig. 6B). This phenotype was observed both in the presence of 10% FCS and after cultivation of the cells for 16 h in serum-free medium. Total lysosomal Ca^2+^ content can be estimated using Gly-Phe-β-naphthylamide (GPN), which as a substrate of the lysosomal protease, cathepsin C, causes osmotic lysis of the acidic compartment. Indeed, also [Ca^2+^]_i_ increases by GPN, both peak increases and AUCs, were significantly enhanced in *Sgpl1*^−/−^-MEFs (Fig. 6B). Interestingly, GPN-induced [Ca^2+^]_i_ increases were significantly delayed in *Sgpl1*^−/−^-MEFs regarding the time from initial [Ca^2+^]_i_ increase until maximum, suggesting that GPN cleavage might be delayed in these cells. Finally, we confirmed that in S1P lyase-deficient MEFs, thapsigargin-induced [Ca^2+^]_i_ increases were augmented (Fig. 6B), similar to our previous measurements in cell suspensions with fura-2 and in single cells with the cameleon Ca^2+^ sensor^19^. Basal [Ca^2+^]_i_, however, could not be determined with FluoForte, because it is not a ratiometric sensor. Taken together, several hallmarks of NPC1 knockout or mutant cells were also observed in S1P lyase-deficient MEFs, with the exception of lysosomal Ca^2+^ release.

### Influence of HDACs

**Figure 7:**
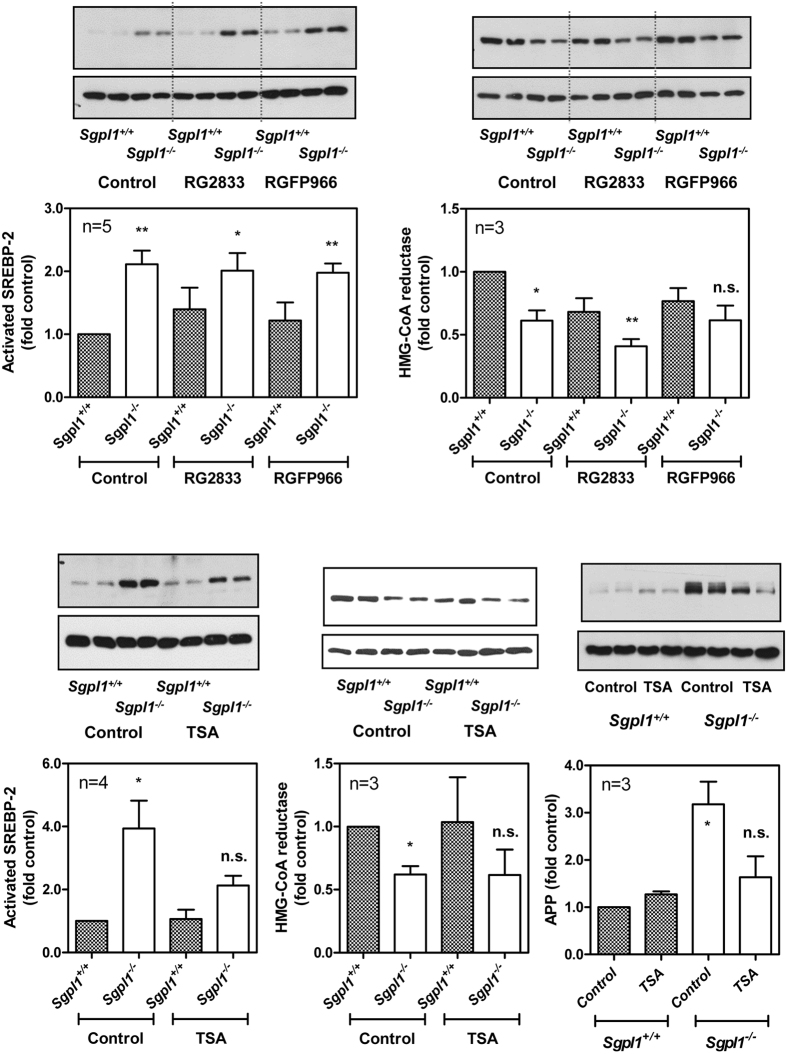
Influence of HDAC inhibitors on activation of SREBP-2 and protein expression of HMG-CoA reductase and APP. The cells were treated with 1 μM RG2833 (HDAC1/3 inhibitor), 2 μM RGFP966 (HDAC3 inhibitor), 1 μM TSA (pan-HDAC inhibitor), or vehicle for 16 h in serum-free medium. Cell lysates were analysed by Western blotting for expression of SREBP-2 activated fragment, HMG-CoA reductase and APP. Shown are representative blots and quantifications with means ± SEM of the indicated number of independent experiments performed in duplicate. *p < 0.05; **p < 0.01; n.s., not significant in one-sample t-test.

We had shown before that HDAC activity and expression of HDAC1 and HDAC3 were decreased in S1P lyase-deficient MEFs, and that HDAC inhibitors imitated while HDAC1/2 overexpression partially alleviated the dysregulation of Ca^2+^ homeostasis in these cells^20^. On the other hand, HDAC inhibitors have been shown to correct the cholesterol trafficking defect in mutant NPC1 fibroblasts, and to be beneficial in animal models of NPC disease^41^,^42^,^43^. Furthermore, there are several reports with controversial observations on the influence of HDACs on regulation of key genes involved in cholesterol homeostasis^44^,^45^. In our cells, the HDAC1/3 inhibitor, RG2833, and the HDAC3 inhibitor, RGFP966, very slightly enhanced SREBP-2 activation and induced HMG-CoA reductase downregulation in wild-type MEFs, but the effects were not significant (Fig. 7). Thus, the selective HDAC1/3 or HDAC3 inhibitors, which target those HDAC isoforms that are downregulated in S1P lyase-deficient MEFs, had the tendency to mimick the *Sgpl1*^−/−^ phenotype in the wild-type MEFs. In contrast, trichostatin A (TSA), the pan-HDAC inhibitor, significantly reduced SREBP-2 activation and APP upregulation in *Sgpl1*^−/−^-MEFs (Fig. 7), in agreement with the studies in NPC1 mutant cells^41^. These results point towards a differential role of HDAC isoforms in S1P lyase-deficient cells.

## Discussion

It has long been known that lack of S1P lyase leads to accumulation of diverse lipids, and therefore, the comparison of S1P lyase deficiency to lipid storage diseases appears reasonable. While the lack of S1P lyase primarily causes accumulation of S1P and sphingosine, an interesting link to cholesterol homeostasis has been discovered by the group of Richard Proia^16^. They showed that in addition to sphingolipids, also phospholipids, triglycerides and cholesterol were elevated in serum and liver of *Sgpl1*^−/−^-mice^16^. In an attempt to understand the link between S1P lyase deficiency and cholesterol homeostasis, we observed in *Sgpl1*^−/−^-MEFs a defect of intracellular cholesterol trafficking, which is a hallmark of NPC. We show here that there are common phenotypic alterations in S1P lyase deficiency and NPC, but also clear differences, which might help to understand the molecular mechanisms involved in this and other lipid storage diseases affecting lysosomal functions.

NPC is a lysosomal lipid storage disease caused by loss-of-function mutations in the NPC1 or NPC2 genes^32^. Clinically, NPC presents with hepatosplenomegaly and progressive neurodegeneration^32^,^46^,^47^,^48^. No causal treatment is available so far, but miglustat, 2-hydroxypropyl-β-cyclodextrin or HDAC inhibitors delay disease progression^42^,^49^,^50^,^51^. Recent data suggest that in addition to NPC1/2 mutations, a pathologic regulation of normal NPC proteins might contribute to human diseases such as atherosclerosis^31^. In this context it is important that the role and function of the NPC genes are only partially understood. Generally, it is thought that free cholesterol derived from endocytosed lipoproteins in late endosomes/lysosomes is bound by the small intraluminal NPC2 protein which hands it over to NPC1^52^,^53^,^54^. NPC1, which is anchored to the outer membrane of late endosomes/lysosomes, then distributes cholesterol to the plasma membrane, Golgi and the ER^30^,^31^. In cells lacking or expressing mutant NPC1 or NPC2, not only cholesterol, but also glycosphingolipids, sphingomyelin and sphingosine accumulate^32^. The accumulation of glycosphingolipids, in particular glucosylceramide, is the rationale for treatment with miglustat. However, the link between intracellular cholesterol transport and accumulation of sphingolipids remains unclear at present. Lloyd-Evans *et al*. have shown that after inhibition of NPC1 with U18666A, accumulation of sphingosine was the earliest event before impairment of lysosomal Ca^2+^ storage and accumulation of cholesterol and glycosphingolipids^38^. Therefore, it has been suggested that sphingosine directly or indirectly affects the function of the NPC proteins^55^. Furthermore, it has been speculated that NPC1 might be required for export of sphingosine, trapped by protonation in acidic compartments, from the lysosomes^56^.

Indeed, sphingosine, besides S1P, accumulates in S1P lyase-deficient fibroblasts. These cells are furthermore characterized by chemoresistance and a disturbed Ca^2+^ homeostasis with elevated basal [Ca^2+^]_i_ and enhanced ER Ca^2+^ storage^17^,^18^,^19^,^20^. Here, we describe the massive disturbance of cholesterol homeostasis in those cells. While cholesterol release was not altered, cholesterol uptake and content were enhanced in the presence of serum. In serum-free conditions, neither uptake nor content of cholesterol were altered, indicating that the source of excess cholesterol was the serum. Since LDL and VLDL receptors were upregulated, it is concluded that the uptake of cholesterol-containing lipoproteins is enhanced in S1P lyase-deficient MEFs, leading to a net increase in cellular cholesterol. In an apparent contradiction, SREBP-2 activation by proteolytic cleavage was enhanced both in the absence and presence of serum, indicating a local lack of cholesterol at the ER. These data point towards a disturbance of intracellular cholesterol trafficking. Indeed, free cholesterol was detected by filipin staining in NPC1-containing vesicles which partially overlapped with lysosomes. Filipin staining of free cholesterol in skin fibroblasts of patients is a standard diagnostic test for NPC^48^,^49^,^57^. Furthermore, increases in amount and processing of SREBP-1 and SREBP-2 have been described in NPC^58^. Other hallmarks of NPC have been described before in S1P lyase-deficiency, without drawing the link to this disease: defective processing of APP, and impaired autophagic flux^36^,^37^,^59^. Here, we show that upregulation of APP and SREBP-2 activation were mimicked by an NPC1 inhibitor in wild-type MEFs. Furthermore, overexpression of NPC1, despite a very low transfection efficiency, reduced APP levels to a level not significantly different from wild-type, proving the involvement of NPC1 in this *Sgpl1*^−/−^ phenotype.

The analysis of cellular sterols in *Sgpl1*^−/−^-MEFs revealed decreased levels of cholesterol precursor molecules, in agreement with downregulation of HMG-CoA reductase, but accumulation of 7α-hydroxycholesterol. The latter lipid can be generated both enzymatically by CYP7A1, and by lipid oxidation^29^. Cellular oxysterols such as 7-ketocholesterol, which can degrade to 7α- and 7β-hydroxycholesterol, are formed in an oxidative cellular environment. Plasma level of 7-ketocholesterol, in particular, has been suggested as a biomarker for NPC^60^, however, this lipid accumulates also in other disorders such as Niemann-Pick disease type B, lysosomal acid lipase deficiency, and Smith-Lemli-Opitz syndrome^61^. Since MEFs usually do not express CYP7A1, it seems likely that 7α-hydroxycholesterol was generated via 7-ketocholesterol by oxidation of cholesterol in *Sgpl1*^−/−^-MEFs, indicating again a similarity to NPC. On the other hand, 24-hydroxycholesterol, exclusively formed enzymatically^28^, was not altered in the knockout cells. The oxysterol accumulation, in return, could have an inhibitory impact on HMG-CoA reductase as described^62^,^63^.

Despite these similarities between S1P lyase deficiency and NPC, we observed an important difference regarding lysosomal Ca^2+^ storage. Pioneering work by the group of Frances Platt has shown that B lymphoblasts and fibroblasts expressing mutant NPC1 contain less Ca^2+^ in the acidic compartment^38^. This appeared as a strong reduction of [Ca^2+^]_i_ increases induced by bafilomycin A1, inhibiting the vesicular H^+^ -ATPase, and GPN, inducing osmotic lysis of the acidic compartment^38^. As mentioned above, inhibition of NPC1 with U18666A caused an initial rapid increase in sphingosine levels, followed only later by lysosomal Ca^2+^ loss and accumulation of lipids. In agreement, extracellular addition of 1–2 μM sphingosine reduced GPN-induced [Ca^2+^]_i_ increases, suggesting that sphingosine released Ca^2+^ from the acidic compartment^38^. In addition, it was recently shown that uncaging of caged sphingosine caused [Ca^2+^]_i_ increases which required the expression of the lysosomal two-pore channel, TPC1, but not TPC2 or mucolipid transient receptor potential channel-1 (TRPML1)^40^. These authors furthermore observed that [Ca^2+^]_i_ increases by uncaging of sphingosine were reduced in NPC human fibroblasts^40^. Contradicting data were published by Shen *et al*.^39^. These authors did not observe an alteration of GPN-induced [Ca^2+^]_i_ increases in *NPC1*^−/−^-CHO cells, but a reduction of TRPML1-mediated lysosomal Ca^2+^ release and currents. This was traced back to an inhibitory effect of the accumulating sphingomyelin on TRPML1^39^. In S1P lyase-deficient MEFs, we observed a significantly and markedly enhanced Ca^2+^ storage, using GPN, and an enhanced Ca^2+^ release by bafilomycin A1. On the background of the present model of sphingosine-induced Ca^2+^ release from lysosomes, this is a particularly interesting observation. As mentioned in the introduction, not only S1P, but also sphingosine accumulated in *Sgpl1*^−/−^-*MEFs*, and while S1P was mainly associated with the nuclei, sphingosine rather accumulated elsewhere in the cells^19^,^20^. Although it is reasonable that sphingosine is trapped by protonation in acidic compartments^56^, the attempts to show a lysosomal localization of sphingosine using fluorescent sphingosine or clickable pac-sphingosine^40^,^64^ have to be regarded with caution because of the rapid metabolism of this lipid. Nevertheless, it is tempting to speculate that sphingosine reaches particularly high levels in the lysosomes, thereby interfering with lysosomal Ca^2+^ storage and/or NPC proteins. Our data however show that while sphingosine might somehow interfere with NPC protein function, its accumulation is not necessarily causing a lysosomal Ca^2+^ loss. Furthermore, we demonstrate that lysosomal cholesterol trapping can occur together with enhanced lysosomal Ca^2+^ storage, indicating that these are two independent events. These results, however, do not preclude that an acute release of sphingosine, e.g., by uncaging of caged sphingosine^40^, causes an immediate Ca^2+^ release from the lysosomes.

The reason for the opposing regulation of lysosomal Ca^2+^ storage in NPC and S1P lyase deficiency remains unclear at present. It has to be kept in mind that aside from the common sphingosine accumulation, the profile of accumulating lipids is not identical in the two conditions. S1P levels are particularly high in S1P lyase deficiency while ceramide and sphingomyelin, dependent on the tissue, are not or only slightly enhanced^16^,^27^,^65^. The ganglioside profile has been analyzed in S1P lyase-deficient neurons, and found to be not altered^27^. In NPC, on the other hand, there is a strong accumulation of sphingomyelin and glycosphingolipids^32^. Thus, while it is speculated here that S1P in S1P lyase-deficient cells and/or sphingomyelin and glycosphingolipids in NPC make the difference, future work would need to compare the lysosomal content of different sphingolipid species in S1P lyase deficiency and NPC in greater detail. Another reason for enhanced lysosomal Ca^2+^ storage in *Sgpl1*^−/−^-MEFs might be the previously described elevated cytosolic [Ca^2+^] and/or the enhanced ER Ca^2+^ storage^19^, both of which might somehow facilitate Ca^2+^ storage in the lysosomes. In this regard it is interesting to note that Lloyd-Evans *et al*. had shown that elevation of cytosolic [Ca^2+^] ameliorated the NPC phenotype in terms of lipid storage, but they did not observe a recovery of the Ca^2+^ phenotype^38^.

The reason for trapping of free cholesterol in *Sgpl1*^−/−^-MEFs also remains unclear at present. According to present paradigms, it is likely that sphingosine accumulation, a common feature of S1P lyase-deficiency and NPC, causes NPC1 dysfunctionality by an unknown mechanism^38^,^55^. However, at least in serum-free conditions, there was a strong difference in NPC1 protein expression between *Sgpl1*^+/+^- and *Sgpl1*^−/−^-MEFs, suggesting that both functionality and expression could be affected in the knockout cells. It is furthermore possible that proteins other than NPC1, indirectly influencing the function of NPC1 or NPC2, mediate the observed effects. Since HDACs obviously play a role in NPC^42^, and since TSA had significant effects on SREBP-2 activation and APP upregulation in the present study, it might be possible that S1P via its influence on HDACs regulates the expression of proteins which are involved in the pathophysiological process.

Finally, the question remains whether the events that we observed in *Sgpl1*^−/−^-MEFs could explain the phenotype of the S1P lyase-deficient mice. However, it has to be regarded that cholesterol homeostasis in cells such as hepatocytes or macrophages is regulated differently from fibroblasts, which e.g. do not express liver X receptor which is a major regulator of NPC1 expression^31^. In hepatocytes from *NPC1*^−/−^-mice, phosphatidylcholine, cholesterol esters, and sphingomyelin were elevated, while triacylglycerol was decreased^66^. These observations were explained by enhanced hepatic cholesterol synthesis at the expense of triglycerides, and resulted in decreased triglyceride levels in *NPC1*^−/−^ mouse serum^67^. In contrast, in the plasma of NPC patients, triglycerides were increased in the majority of patients, while LDL and HDL cholesterol was decreased^68^. Thus, the mechanisms regulating liver and plasma lipids in NPC1 deficiency and dysfunction are complex and differ between mouse and human. Therefore, the situation with elevated cholesterol, triglycerides and phospholipids in serum and liver of *Sgpl1*^−/−^-mice^16^ cannot be explained by the present data and remains to be analyzed in greater detail.

In summary, S1P lyase-deficient MEFs share many features of NPC1/2 knockout or NPC patient fibroblasts, in particular, the cholesterol trafficking defect and altered APP processing/upregulation, while lysosomal Ca^2+^ homeostasis is altered in an opposing manner. These observations first of all show that a primary accumulation of S1P and sphingosine, as observed in *Sgpl1*^−/−^-MEFs, suffices to induce an impairment of cholesterol trafficking. Secondly, it is shown for the first time that lysosomal lipid accumulation and Ca^2+^ storage are regulated independently of each other, and that Ca^2+^ release from lysosomes by accumulating sphingosine cannot explain the phenotype of S1P lyase-deficient MEFs. Nevertheless, it remains unclear how the sphingoid bases regulate NPC proteins, directly or indirectly, and how lysosomal Ca^2+^ storage is regulated. Our results thus add to the complexity of observations but may also help to obtain a better understanding of cellular cholesterol and lysosomal Ca^2+^ homeostasis.

## Materials and Methods

### Materials

RG2833 and RGFP966 were purchased from Selleck Chemicals LLC (Houston, TX, USA). U18666A and GPN were obtained from Cayman Chemical Company (Ann Arbor, MI, USA). LysoTracker Red DND-99, LysoSensor Yellow/Blue DND-160 and Hoechst 33342 were from Molecular Probes/Invitrogen (Thermo Fisher Scientific, Darmstadt, Germany). GFP-certified FluoForte was from Enzo Life Sciences GmbH (Lörrach, Germany). Filipin III from *streptomyces filipinensis, N*,*N*-dimethylformamide and ApoA-I were purchased from Sigma-Aldrich Chemie GmbH (Taufkirchen, Germany). Bafilomycin A1 was obtained either from Calbiochem/Merck Millipore (Darmstadt, Germany) or from Tocris Bioscience (Bristol, UK). Fatty acid-free BSA was from PAA Laboratories (Pasching, Austria). [1,2-^3^H(N)]Cholesterol (1.813 TBq/mmol) was from PerkinElmer LAS (Rodgau, Germany). All other chemicals were from previously described sources^18^,^20^. The mouse NPC1-His6-EYFP expression plasmid (NPC1-YFP; Addgene plasmid 53523) was kindly provided by Dr. Matthew P. Scott via Addgene^69^.

### Cell culture and transfection

If not stated otherwise, all cell culture media, buffer and supplements were supplied by Gibco/Thermo Fisher Scientific (Darmstadt, Germany). Embryonic fibroblasts from S1P lyase-deficient and corresponding wild-type mice (*Sgpl1*^+/+^-and *Sgpl1*^−/−^-MEFs) had kindly been provided by Dr. Paul P. Van Veldhoven (Leuven, Belgium). The cells were cultured as described previously in DMEM/F12 supplemented with 100 U/ml penicillin G, 0.1 mg/ml streptomycin, and 10% FCS in a humidified atmosphere of 5% CO_2_/95% air at 37 °C^18^,^20^. For serum-free cell culture, the cells were grown in fibroblast growth medium supplemented with 1 ng/ml basic fibroblast growth factor and 5 μg/ml insulin (PromoCell, Heidelberg, Germany). Transfection was performed with either the GeneJuice transfection agent (Novagen/EMD Millipore Corporation, Billerica, MA, USA) or by electroporation. For electroporation, the Nucleofector II device and the Amaxa MEF1 Nucleofector kit were used (Lonza, Basel, Switzerland). The cells were seeded onto 6 cm-dishes and grown to near confluence. The cells were washed with 1 ml of Dulbecco’s phosphate-buffered saline (DPBS) and detached with trypsin/EDTA. After centrifugation at 100× g for 5 min, the pellet was resuspended in 100 μl MEF1 Nucleofector solution. Then, 5 μg of plasmid DNA were added, the mixture was transferred into the electroporation cuvette, and program A-23 of the Nucleofector II device was applied. Thereafter, 500 μl of pre-warmed growth medium was added and the cells were seeded onto 8-well chambered coverslides (μ-slide; ibidi GmbH, Martinsried, Germany) coated with poly-L-lysine.

### PCR

mRNA was isolated with TRIZOL (Sigma-Aldrich Chemie GmbH, Taufkirchen, Germany). cDNA was prepared with the RevertAid first strand cDNA synthesis kit (Fermentas, St. Leon-Rot, Germany). Real-time PCR was performed with the Applied Biosystems 7500 Fast Real-Time PCR System. Probes, primers, and the reporter dyes 6-FAM and VIC were from Applied Biosystems (Darmstadt, Germany). The cycling conditions were 50 °C for 5 s (1 cycle) 95 °C for 15 min (1 cycle), followed by 95 °C for 15 s and 60 °C for 1 min (40 cycles). mRNA expression levels were analyzed by the ΔΔCt method with GAPDH as reference. Expression of the VLDL receptor was analyzed by normal PCR (Taq polymerase from Thermo Fisher Scientific, Darmstadt, Germany) with the following primer pair: forward, 5′-GTGGACTGGTTCCTGGAGGGAT-3′; reverse, 5′-AAGGAGACTTCAGCGCTGGCT-3′. 100 ng cDNA were subjected to the following PCR program: 5 min at 94 °C, then 45 cycles of 30 s at 94 °C, 1 min at 60 °C, 1 min 72 °C, then 7 min at 72 °C.

### Western blotting

Cells grown to near confluence on 6 cm-dishes were lysed, separated by SDS gel electrophoresis and blotted onto polyvinylidene difluoride membranes. Antibodies directed against Abca1 (ab18180), LDL receptor (ab30532), HMG-CoA reductase (ab174830), SREBP-2 active fragment (ab30682), NPC1 (ab134113), and APP (ab32136) were obtained from Abcam (Cambridge, UK). Antibodies to AMPKα (#2603 S) and phospho-Thr172-AMPKα (#2535 S) were from Cell Signaling Technology (Danvers, MA, USA), while anti-β-actin (A5441) was from Sigma-Aldrich Chemie GmbH (Taufkirchen, Germany). HRP-conjugated secondary antibodies were from GE Healthcare (Freiburg, Germany). The enhanced chemiluminescence system was from Millipore Corporation (Billerica, MA, USA).

### Measurement of cholesterol release and uptake

For measurements of cellular cholesterol release, the cells were seeded onto 3.5 cm-dishes and labelled for 24 h with DMEM/F12 medium supplemented with 10% FCS and 0.025 μCi/ml [^3^H]cholesterol. Thereafter, the cells were washed twice with DMEM/F12 supplemented with 1% BSA, followed by incubation for 6 h or 24 h, respectively, in DMEM/F12 containing 1% BSA and 250 ng/ml ApoA-I. The supernatants were collected, supplemented with scintillation cocktail (IRGA-SAFE PLUS; PerkinElmer LAS, Rodgau, Germany), and radioactivity was measured by liquid scintillation counting. For measurements of cellular cholesterol uptake, cells grown on 3.5 cm-dishes were washed twice with DMEM/F12 containing either 10% FCS (medium A) or 1% BSA (medium B). Then, the cells were incubated for 2 h with medium A or medium B supplemented with 0.1 μCi/ml [^3^H]cholesterol. Cell monolayers were washed twice with DPBS and lysed with 1 ml 0.1 M NaOH per dish. Scintillation cocktail was added and radioactivity was quantified by liquid scintillation counting. Separate dishes were used for protein measurements.

### Measurement of cellular cholesterol, non-cholesterol sterol and oxysterol content

In a first series of experiments, cellular cholesterol content was measured with the Amplex Red cholesterol assay kit (Molecular Probes/Invitrogen; Thermo Fisher Scientific, Darmstadt, Germany). Briefly, cells seeded onto 6 cm-dishes and grown to near confluence were washed with DPBS and lysed with 200 μl 1× reaction buffer for 30 minutes on ice. After centrifugation at 16,200× g for 10 min, supernatants were collected and diluted to 1 mg/ml protein with 1× reaction buffer. 50 μl of each sample was complemented with 50 μl of working solution and incubated for 30 min at 37 °C, protected from light. The fluorescence was measured in a fluorescence microplate reader at 540 nm excitation and 590 nm emission wavelengths.

In a second series of experiments, cholesterol, non-cholesterol sterol and oxysterol content was analyzed by GC. Cholesterol, its precursors lanosterol, lathosterol, and desmosterol, and the oxysterol metabolites 7α-, 24-, and 27-hydroxycholesterol were extracted from cell pellets by chloroform-methanol and determined after derivatization to the corresponding trimethylsilyl ethers by GC-flame ionization detection (GC-FID for cholesterol, HP6890 Series GC-System, Agilent Technologies, Waldbronn, Germany) and GC-mass spectrometry detector-selected ion monitoring (GC-MSD-SIM for sterols and oxysterols except cholesterol; Agilent Technologies 6890 Network GC coupled with an Agilent Technologies 5975B inert MSD, Agilent Technologies, Waldbronn, Germany) as reported previously^26^,^27^.

### Filipin staining

1 mg filipin III was dissolved in 666 μl *N*,*N*-dimethylformamide and added to 33.33 ml DPBS. Cells grown on poly-L-lysine-coated 8-well chambered coverslides were washed twice with 200 μl ice-cold DPBS and fixed for 1 h with 200 μl 4% paraformaldehyde on ice. After washing three times with 200 μl DPBS, the cells were stained with 200 μl filipin III solution for 1 h at room temperature. Then, they were washed again three times with 300 μl DPBS and kept in the dark at 4 °C until microscopic analysis. For double staining with lysotracker, the cells were first incubated with 75 nM LysoTracker Red DND-99 in serum-free medium for 45 min before fixing with paraformaldehyde and staining with filipin.

### Fluorescence microscopy

Confocal laser scanning microscopy was performed with a Zeiss LSM510 Meta system equipped with an inverted Observer Z1 microscope and a Plan-Apochromat 63 × /1.4 oil immersion objective (Carl Zeiss MicroImaging GmbH, Göttingen, Germany). The following excitation (ex) laserlines and emission (em) filter sets were used: Hoechst 33342: ex 405 nm, em band-pass 420–480 nm; filipin: ex 405 nm, em long-pass 420 nm; filipin (with YFP): ex 405 nm, em band-pass 420–480 nm; LysoTracker Red DND-99: ex 561, em band pass 575–630; NPC1-YFP: ex 514 nm, em long-pass 530 nm.

### Measurements of lysosomal pH

Relative measurements of lysosomal pH were performed with the acidotropic and ratiometric pH sensor, LysoSensor Yellow/Blue DND-160. MEFs kept for 16 h in serum-free medium were detached with trypsin and loaded with 100 nM LysoSensor Yellow/Blue for 30 min at room temperature in Hank’s balanced salt solution (HBSS; 118 mM NaCl, 5 mM KCl, 1 mM CaCl_2_, 1 mM MgCl_2_, 5 mM D-glucose and 15 mM HEPES pH 7.4). Thereafter, the cells were washed with HBSS and resuspended at a density of ∼1 × 10^6^ cells/ml. Fluorescence measurements were performed in cell suspensions in a Hitachi F2500 spectrofluorometer at room temperature. Excitation was altered between 329 and 384 nm while emission was recorded at 490 nm. At excitation = 329 nm, the fluorescence of LysoSensor Yellow/Blue DND-160 decreases with increasing proton concentrations, while at excitation = 384 nm, its fluorescence increases with increasing proton concentrations; thus, lower values of ratio 329/384 represent more acidic pH.

### [Ca^2+^]_i_ measurements

MEFs grown on 8-well chambered coverslides were loaded with 1 μM GFP-certified FluoForte in HBSS for ~45 min at room temperature. Thereafter, they were analyzed by microscopy using the above mentioned Zeiss LSM510 Meta system and the Plan-Apochromat 63×/1.4 oil immersion objective. For excitation, the 543 nm laser line was used while emission was recorded with a 570–615 nm band pass filter or in channel S at 560–700 nm. Time series were generated with 1 image/s, and mean fluorescence of single cells was monitored by defining appropriate regions of interest.

### Data analysis and presentation

Averaged data are means ± SEM from the indicated number (n) of independent experiments. For quantitative evaluation of Western blots, bands were analyzed with ImageJ (http://imagej.nih.gov), normalized to β-actin and expressed as fold of wild-type control. Graphical presentations and statistical analyses were performed with Prism-5 (GraphPad Software, San Diego, CA). Microscopic images were analyzed and presented with the ZEN software (Carl Zeiss MicroImaging GmbH, Göttingen, Germany). For quantification of [Ca^2+^]_i_ measurements, the fluorescence at a given time point was normalized to baseline fluorescence, defined as mean fluorescence during the first ~15–20 s (F/F_0_). Maximal increases in fluorescence after stimulation (ΔF/F_0_) and areas under the curve (AUCs) were calculated with Prism-5 after Savitsky-Golay smoothing of the curves by averaging each data point with its 2 preceding and subsequent data points. AUC measurements were performed by integrating the area between F_0_ and F at all time points after stimulation until return to baseline or up to a defined end point. Time to peak was defined as time (s) from start of a [Ca^2+^]_i_ increase to the maximal [Ca^2+^]_i_ increase.

## Additional Information

**How to cite this article:** Vienken, H. *et al*. Characterization of cholesterol homeostasis in sphingosine-1-phosphate lyase-deficient fibroblasts reveals a Niemann-Pick disease type C-like phenotype with enhanced lysosomal Ca^2+^ storage. *Sci. Rep.* **7**, 43575; doi: 10.1038/srep43575 (2017).

**Publisher's note:** Springer Nature remains neutral with regard to jurisdictional claims in published maps and institutional affiliations.

## Supplementary Material

Supplemental Figures
