# Supplementary material for: Characterization of cholesterol homeostasis in sphingosine-1-phosphate lyase-deficient fibroblasts reveals a Niemann-Pick disease type C-like phenotype with enhanced lysosomal Ca2+ storage
Source: Sci Rep. 2017 Mar 6;7:43575. doi: 10.1038/srep43575 (PMC5337937; doi:10.1038/srep43575)
Supplement: Supplemental Figures [file srep43575-s1.pdf]

SUPPLEMENTAL FIGURES

Characterization of cholesterol homeostasis in sphingosine-1-phosphate lyase-deficient fibroblasts reveals a Niemann-Pick disease type C-like phenotype with enhanced lysosomal Ca^{2+} storage

Hans Vienken[1], Nathalie Mabrouki[1], Katja Grabau[1], Ralf Frederik Claas[1], Agnes Rudowski[1], Nina Schömel[1], Josef Pfeilschifter[1], Dieter Lütjohann[2], Gerhild van Echten-Deckert[3], and Dagmar Meyer zu Heringdorf[1,*]

[1]Institut für Allgemeine Pharmakologie und Toxikologie, Klinikum der Goethe-Universität, Frankfurt am Main, Germany;

[2]Institut für Klinische Chemie und Klinische Pharmakologie, Universitätsklinikum Bonn, Germany;

[3]Membranbiologie und Lipidbiochemie, Einheit des Life and Medical Sciences (LIMES) Instituts, Universität Bonn, Germany

*Corresponding author at: Institut für Allgemeine Pharmakologie und Toxikologie, Klinikum der Goethe-Universität, Theodor-Stern-Kai 7, 60590 Frankfurt am Main, Germany. Tel.: +49-69-6301-3906; Fax: +49-69-6301-7942

E-mail address: heringdorf@med.uni-frankfurt.de (D. Meyer zu Heringdorf)

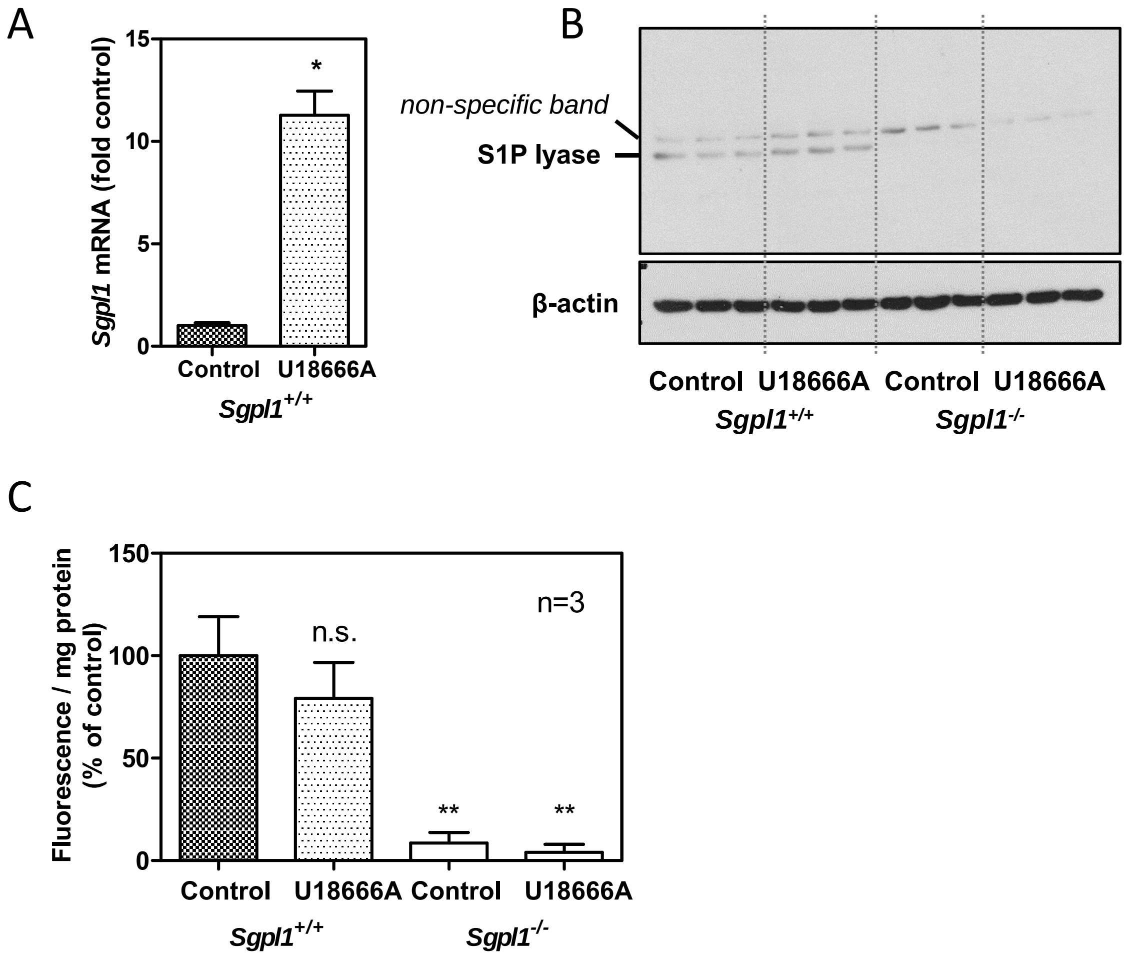

Supplemental Figure 1: Regulation of S1P lyase expression and activity by the NPC1 inhibitor, U18666A. A, Sgpl1 mRNA expression was analyzed by quantitative PCR (means±SD; representative experiment; n=3). B, S1P lyase protein levels were determined by Western blotting (representative experiment). C, S1P lyase activity was measured using the previously described fluorogenic substrate (means±SEM; n=3 independent experiments performed in triplicate). The cells had been incubated with or without 25 µM U18666A in serum-free medium for 16 h. *, $p<0.05$; **, $p<0.01$; n.s., not significant in one-sample t-test.

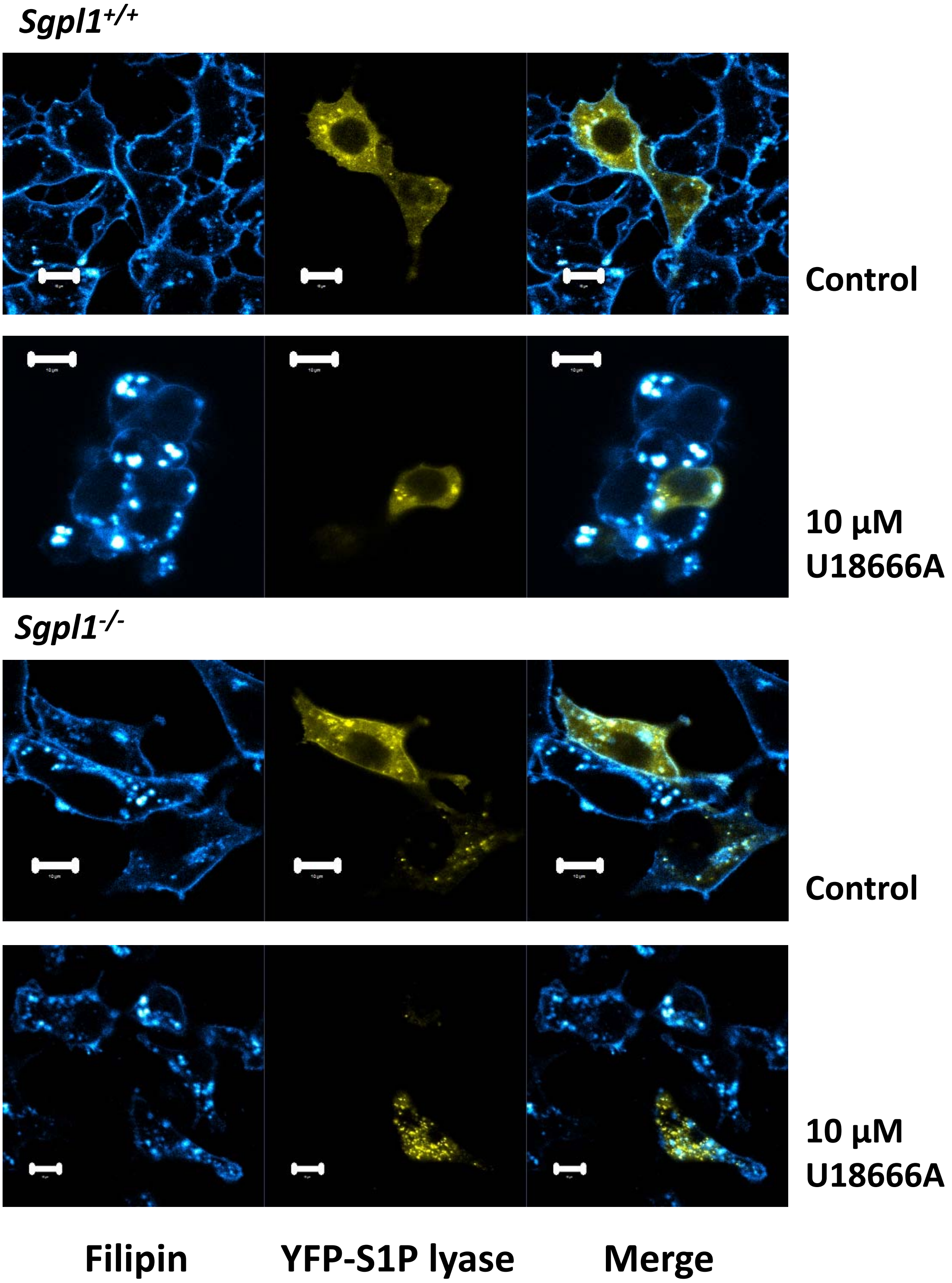

Supplemental Figure 2: No effect of S1P lyase overexpression on U18666A-induced cholesterol sequestration. The cells had been incubated without or with 10 µM U18666A for 16 h in medium containing 10 % FCS. Bars, 10 µm.

Materials and Methods to Supplemental Figures

The Taqman primer probeset for *Sgpl1* (Mm00486079_m1) was from Applied Biosystems (Darmstadt, Germany). The S1P lyase antibody (HPA021125) was from Sigma-Aldrich Chemie GmbH (Taufkirchen, Germany). The S1P lyase activity assay was performed as described before[1]. Briefly, *Sgpl1*$^{+/+}$- and *Sgpl1*$^{-/-}$-MEFs were incubated for 16 h with or without 25 µM U18666A in serum-free medium. Thereafter, the cells were detached, washed with 500 µM potassium phosphate buffer pH 7.4 with or without 25 µM U18666A, and lysed by three cycles of freeze-thawing. 75 µl of cell lysate (10 mg/ml) were incubated with 5 µl 0.5 mM Na_3VO_4, 5 µl 5 mM pyridoxal phosphate, 5 µl solvent or U18666A to yield a final concentration of 25 µM, and 10 µl of 1.25 mM S1P lyase fluorogenic substrate (Cayman Chemical Company, Ann Arbor, MI, USA). After incubation for 6 h at 37 °C in the dark, the reaction was stopped by addition of 50 µl methanol. After additional incubation for 2 h, fluorescence was measured using excitation and emission wavelengths of 355 nm and 460 nm, respectively. The respective buffers without cell lysate were used as blanks. The plasmid for expression of YFP-tagged S1P lyase (Source BioScience LifeSciences expression clone IOH28907 in pdEYFP-C1amp vector) has been described before[2]. Transfection of MEFs with YFP-tagged S1P lyase was performed with the GeneJuice transfection agent as described in the main body of the manuscript. The cells were treated with or without 10 µM U18666A for 16 h in medium containing 10 % FCS. Filipin staining was performed as described in the main body of the manuscript. Microscopic settings were identical with those in Fig. 4C showing filipin staining in combination with NPC1-YFP fluorescence.

References to Supplemental Figures

1. Bedia, C. *et al.* Synthesis of a fluorogenic analogue of sphingosine-1-phosphate and its use to determine sphingosine-1-phosphate lyase activity. *Chembiochem: a European journal of chemical biology* **10,** 820–822 (2009).
2. Ihlefeld, K., Claas, R. F., Koch, A., Pfeilschifter, J. M. & Meyer zu Heringdorf, D. Evidence for a link between histone deacetylation and Ca^{2+} homoeostasis in sphingosine-1-phosphate lyase-deficient fibroblasts. *Biochem. J.* **447,** 457–464 (2012).
